# Effects of cutting time and maceration on preference and nitrogen balance in beef steers fed mixed birdsfoot trefoil–timothy grass hay cut at sunrise or sundown

**DOI:** 10.1093/tas/txaa168

**Published:** 2020-09-10

**Authors:** Luiz H P Silva, André F Brito, Carole Lafrenière, Robert Berthiaume

**Affiliations:** 1 Department of Agriculture, Nutrition, and Food Systems, University of New Hampshire, Durham, NH; 2 Agricultural Research Station, Université du Québec en Abitibi-Témiscamingue, Notre-Dame-du-Nord, Canada; 3 Private Consultant, Expert in Forage Systems, Sherbrooke, Canada

**Keywords:** beef cattle, diurnal cutting management, nonstructural carbohydrates, sucrose

## Abstract

Forages cut at sundown usually contain a greater concentration of nonstructural carbohydrates (NSC) than those cut at sunrise. Maceration can speed up the rate of moisture loss of cut forage during field drying and reduce NSC utilization by plant cells. We aimed to evaluate the effects of cutting time and forage maceration on feed preference, apparent total tract digestibility of nutrients, and N balance in growing steers. A mixed sward of birdsfoot trefoil and timothy grass was divided into two halves, with the first half cut at sundown (1800 h) after a sunny day and the second half at sunrise (0600 h) the next day. Approximately 50% of the sundown- and sunrise-cut herbage were macerated. Forages were harvested as hay resulting in four treatments: 1) sunrise-cut hay (AM); 2) AM plus maceration (AM-M); 3) sundown-cut hay (PM); and 4) PM plus maceration (PM-M). Hays were offered as the sole feed source to four crossbred steers (296.1 ± 7.25 kg) according to a 4 × 4 Latin square design with a 2 × 2 factorial arrangement of treatments. Each period lasted 21 d with 14 d for diet adaptation and 7 d for collection. Hays cut at sundown had 15% greater NSC than those cut at sunrise. A cutting time by maceration interaction was found (*P* < 0.05) for intake and apparent digestibility of crude protein (CP), indicating that these two variables decreased more when maceration was applied to sundown- versus sunrise-cut hays. Similarly, interaction effects were observed (*P* < 0.05) for total digestible nutrients and digestible energy, showing that maceration decreased the energetic value of sundown-cut hays but did not change that of sunrise-cut hays. Steers fed hays cut at sundown had decreased urinary N excretion and improved retained N (*P* < 0.05), whereas N retention was reduced by maceration (*P* < 0.05). In addition, six crossbred steers were used to assess feed preference, 2 wk before (period 1) and 1 wk after (period 2) the digestibility trial. Animals were randomly assigned to receive a sequence of the four hays combined in pairs. The intake rate was greater for sundown- than sunrise-cut hays, and it was decreased by maceration. Steers showed the greatest preference for PM hay, while AM-M was the most rejected. In conclusion, shifting forage cutting from sunrise to sundown increased hay NSC concentration, which resulted in improved N utilization and preference. Forage maceration during field drying decreased CP concentration and N retention in beef steers under the conditions of our study.

## INTRODUCTION

The rate of carbon assimilation by photosynthetically active plant tissues can exceed the carbon exportation rate during a sunny day leading to the accumulation of nonstructural carbohydrates (NSC; Bertrand et al., 2008; De Oliveira et al., 2018). Therefore, the concentration of NSC in fresh forage is usually greater at sundown than sunrise (Brito et al., 2016). However, plant enzymes remain active during field drying so that they can hydrolyze intact proteins into nonprotein N (Cavallarin et al., 2005). Field drying also reduces NSC concentration through cell respiration (Rees, 1982). Maceration may improve hay quality by speeding up herbage dehydration, which can decrease the catabolism of carbohydrates and proteins in plant tissues (Savoie, 2001). Brito et al. (2008) reported that alfalfa cut at sundown and harvested as baleage had greater apparent total tract digestibility of organic matter (OM) than baleage cut at sunrise. Huntington and Burns (2007) demonstrated that sundown-cut grass hay was preferred over its sunrise-cut counterpart by beef steers. In our companion dual-flow continuous culture study, ruminal NH_3_-N concentration was reduced in fermenters dosed with mixed birdsfoot trefoil (BFT; *Lotus corniculatus* L.) and timothy grass (*Phleum pretense*) hay cut at sundown (Kokko et al., 2013). In addition, an interaction was observed whereby maceration decreased true dry matter (DM) digestibility in sundown- but not in sunrise-cut hay (Kokko et al., 2013). These previous results need to be validated and reconciled in vivo to further assess the potential interaction of diurnal cutting time and maceration on N utilization and feed preference. We hypothesized that the concentration of NSC would increase by shifting forage cutting from sunrise to sundown and that maceration could interact with cutting time to improve hay nutritive value, N use efficiency, and feed preference in beef steers. Our objective was to evaluate the effects of cutting time and maceration on nutrient digestibility, N balance, and preference in beef steers fed mixed BFT–timothy grass hay as the sole feed source.

## MATERIALS AND METHODS

Care and handling of the animals were conducted as outlined in the guidelines of the Canadian Council on Animal Care (1993), and the study was approved by the Institutional Animal Care Committee of the Dairy and Swine Research and Development Centre (protocol number K-152).

### Forage Harvesting

Forages were harvested as hay from a mixed BFT–timothy grass stand in Kapuskasing, ON, Canada (49°24´ N, 82°26´ W, 218 m above sea level). The field used had 2 ha, with the legume-grass forage mix established for approximately 4 yr. At the time of harvest, BFT and timothy grass were at the early flowering and fully headed stages of development, respectively. The stand had a botanical composition (DM basis) of 43% BFT (cv. Bull), 53% timothy grass (cv. Richmond), and 4% weeds determined by hand separation (Lafrenière and Drapeau, 2011). Forages were harvested as hay, resulting in four treatments: 1) sunrise-cut hay (AM); 2) AM plus maceration (AM-M); 3) sundown-cut hay (PM); and 4) PM plus maceration (PM-M). Sundown-cut treatments (PM and PM-M) were harvested at 1800 h (11.5 h after sunrise) following a sunny day on August 19, 2008 (average daytime temperature: 16.1 °C, humidity: 73.1%), while AM and AM-M treatments were harvested at 0600 h (before sunrise) on August 20, 2008 (average daytime temperature: 21.7 °C, humidity: 71.5%). Forages were cut with a Hesston model 1160 conventional mower-conditioner (AGCO, Duluth, GA). Both nonmacerated treatments were tedded three times (at 1300 h on August 20, 2008, and at 0900 and 1300 h on August 21, 2008) with a Ziegler model HR551 tedder (Shakopee, MN) and left on the field to dry until baling with a Massey Ferguson model 9 square baler (AGCO, Duluth, GA). Macerated treatments were mechanically macerated (Macerator 6610; AgLand Industries, Arborg, MB, Canada) 4 h (AM-M) and 12 h (PM-M) after cutting and tedded three times (1300 h on August 20, 2008, and 0900 and 1300 h on August 21, 2008) before baling. All treatments were baled between 1500 and 1900 h on August 21, 2008, and stored inside a barn until feeding. Baling was done following the sequence PM-M, AM-M, PM, and AM because we were expecting increased moisture losses in response to maceration. No rainfall events occurred during field drying. Three samples of fresh forage from each treatment were obtained immediately after mowing and before maceration, tedding, and baling. These samples were dried, pooled, and finally ground for chemical analysis as described below. The chemical composition of fresh and conserved mixed BFT–timothy grass forages are presented in Tables 1 and 2, respectively.

**Table 1.** Chemical composition (% of DM, unless otherwise noted) of mixed BFT–timothy herbage^*a*^ at mowing, maceration, tedding, and baling

	Treatment^*b*^			
Item	AM	AM-M	PM	PM-M
Mowing				
DM, % of fresh matter	18.3	18.6	23.2	23.4
CP	14.8	13.8	13.7	13.9
ADF	33.1	33.9	31.6	31.7
aNDFom	53.3	54.8	53.3	53.4
WSC	9.82	9.77	12.0	13.2
TESC	10.3	9.39	10.8	11.5
Starch	0.86	1.03	1.49	1.48
NSC	11.1	10.4	12.3	13.0
Maceration				
DM, % of fresh matter	–	21.3	–	23.1
CP	–	13.9	–	13.1
ADF	–	34.3	–	33.5
aNDFom	–	54.3	–	54.9
WSC	–	10.7	–	12.3
TESC	–	9.39	–	10.8
Starch	–	0.89	–	0.74
NSC	–	10.3	–	11.6
Tedding				
DM, % of fresh matter	34.1	41.4	38.6	43.0
CP	13.7	13.2	14.1	13.3
ADF	34.1	37.3	32.4	33.0
aNDFom	56.2	58.5	52.3	54.8
WSC	9.87	9.67	12.4	12.0
TESC	9.16	9.37	11.7	10.9
Starch	0.54	0.55	0.61	0.73
NSC	9.70	9.92	12.3	11.6
Baling				
DM, % of fresh matter	72.9	78.1	72.9	78.8
CP	11.4	11.2	12.1	11.5
ADF	36.2	36.8	35.5	37.4
aNDFom	60.5	61.5	57.3	61.3
WSC	9.62	9.68	11.8	11.4
TESC	8.34	8.29	10.5	9.25
Starch	0.43	0.50	0.50	0.56
NSC	8.76	8.79	11.0	9.81

^*a*^Values from pooled samples of each treatment (n = 3).

^*b*^AM = sunrise cut, nonmacerated; AM-M = sunrise cut, macerated; PM = sundown cut, nonmacerated; PM-M = sundown cut, macerated.

**Table 2.** Chemical composition of mixed BFT–timothy grass hays^*a*^ fed to beef steers (mean ± SD)

	Treatment^*b*^			
Item	AM	AM-M	PM	PM-M
DM, % of fresh matter	85.5 ± 2.37	85.9 ± 1.96	84.8 ± 2.81	86.0 ± 2.00
	^___________________________________________________________________^% of DM^___________________________________________________________________^			
OM	92.3 ± 0.57	92.4 ± 0.57	92.1 ± 0.52	92.5 ± 0.48
EE	2.22 ± 0.13	2.06 ± 0.17	2.33 ± 0.20	2.10 ± 0.22
CP	11.5 ± 0.72	10.9 ± 0.65	12.0 ± 0.68	10.8 ± 0.82
Soluble CP, % of CP	23.6 ± 1.48	27.0 ± 1.55	26.0 ± 2.42	27.5 ± 2.50
ADF	39.9 ± 1.61	40.1 ± 0.75	37.2 ± 2.15	38.9 ± 2.27
aNDFom	63.4 ± 2.54	64.1 ± 1.28	59.2 ± 4.05	61.1 ± 2.66
ADICP	0.66 ± 0.29	0.57 ± 0.20	0.60 ± 0.21	0.56 ± 0.14
NDICP	4.05 ± 0.46	3.80 ± 0.22	3.86 ± 0.48	3.61 ± 0.37
TESC	7.79 ± 0.88	8.47 ± 0.86	9.30 ± 1.69	9.43 ± 1.09
Starch	0.41 ± 0.04	0.45 ± 0.06	0.45 ± 0.06	0.51 ± 0.07
NDSF^*c*^	3.32 ± 2.09	3.74 ± 1.77	4.50 ± 2.44	3.82 ± 1.39
Organic acids^*d*^	7.87 ± 1.54	6.97 ± 1.29	9.58 ± 1.32	8.67 ± 1.83
NSC^*e*^	8.20 ± 0.87	8.91 ± 0.86	9.75 ± 1.70	9.94 ± 1.09
NFC^*f*^	19.2 ± 1.90	19.1 ± 1.22	23.4 ± 3.92	22.2 ± 2.05

^*a*^Means obtained from weekly samples throughout the digestibility and N balance trial and daily samples throughout the feed preference trial (*n* = 16), except for soluble protein, which were analyzed at samples from the third week of the digestibility trial (*n* = 4).

^*b*^AM = sunrise cut, nonmacerated; AM-M = sunrise cut, macerated; PM = sundown cut, nonmacerated; PM-M = sundown cut, macerated.

^*c*^Calculated according to Hall et al. (1999).

^*d*^Calculated according to Hall et al. (1999).

^*e*^TESC + starch.

^*f*^NFC = 100 − [CP + EE + (aNDFom − NDICP) + ash].

### Digestibility and Nitrogen Balance Trial

Four growing crossbred Angus steers averaging 296.1 ± 7.25 kg of body weight (BW) and 8.3 ± 0.53 mo old were used. Steers were randomly assigned to a 4 × 4 Latin square design with a 2 × 2 factorial arrangement of treatments. Treatments consisted of the combination of two cutting times, macerated or not after cutting. One week prior to the start of the digestibility and N balance trial, steers were adapted to steel crates (2.5 m length × 1.60 m width) fitted with a swivel stanchion allowing free access to water and a front tip-down manger providing easy access to the animals and feeding. Each experimental period lasted 21 d with 14 d for diet adaptation and 7 d for data and sample collection. During the first week of each experimental period, steers were fed ad libitum to determine voluntary feed intake. About 90% of the total voluntary feed intake was fed in the second and third weeks to avoid orts during the sampling week and the confounding effect of potential differences in DM intake (DMI) on nutrient digestibility. Bales were chopped prior to feeding using a hydraulic TMR mixer (model 400; Supreme International Ltd., Wetaskiwin, AB, Canada) equipped with knives to keep hay particles within 8–12 cm length and minimizing leaf losses.

Steers were fed each source of hay once daily at 0800 h. Seventy grams of a mineral–vitamin mix (containing 120 g sodium, 114 g calcium, 60 g phosphorus, 3.6 g sulfur, 2.7 g magnesium, 0.5 g potassium, 398 mg iron, 369 mg copper, 48 mg zinc, 35.1 mg cobalt, 24 mg manganese, 10 mg selenium, 2.1 mg iodine, and 5,029 IU vitamin E per kilogram) was top dressed on hay daily. Samples of each hay were collected daily and kept at room temperature. Next, hay samples were pooled by week, dried in a forced-air oven at 55 °C for 48 h, ground through a 1-mm screen using a Wiley mill (Arthur H. Thomas Co., Philadelphia, PA), and stored in zip-lock plastic bags at room temperature until analyzed. Feed intake was considered equal to hay offered because orts were avoided during the sampling week (i.e., from day 15 to 21) of each experimental period.

Total collection of feces and urine were done throughout the last week of each experimental period. Urine was collected using a harness and funnel fitted to the belly of each steer. The funnel was connected to a line that transported urine to a stainless-steel container filled with concentrated H_2_SO_4_ (18 M; 25 mL of H_2_SO_4_/kg of urine). Feces were recovered from each steer through a plastic-lined wooden box located over the gutter. At the end of each collection day, total acidified urine and feces were weighed. Daily collected urine was mixed and approximately 5% of the total fresh weight was placed in a freezer (−20 °C). This procedure was repeated daily, resulting in composited urine samples that were kept frozen (−20 °C) until analyzed. Daily collected fecal samples were homogenized and approximately 5% of the total fresh weight was subsampled and placed in a freezer (−20 °C). These daily fecal subsamples were composited, oven-dried (55 °C), ground through a 1-mm screen using a Wiley mill (Arthur H. Thomas Co.), and finally stored in zip-lock plastic bags at room temperature until analyzed.

### Feed Preference Trial

Two feed preference trials were performed starting 2 wk before and 1 wk after the completion of the digestibility and N balance experiment, respectively. Six growing crossbred Angus steers were used in the feed preference trials, including four animals also used in the digestibility and N balance phase. Steers averaged 271.7 ± 7.24 kg of BW and 6.9 ± 0.5 mo of age at the beginning of the first feed preference trial and 353.7 ± 9.77 kg of BW and 11.5 ± 0.5 mo of age at the beginning of the second feed preference trial. Steers were housed individually in tie stalls with concrete floors covered with rubber mats. Each stall was equipped with an individual wooden manger split in half to accommodate individual pairs of hay combinations as described below and an automatic drinker for ad libitum intake of water. During the adaptation period (4 d), each steer was fed randomly one of the four hays ad libitum daily in order to allow animals to associate individual hays with postingestive metabolic “feelings” and taste (Fisher et al., 2002). Therefore, at the end of this 4-d adaptation period, all steers had been given the opportunity to consume all four hays (i.e., AM, AM-M, PM, and PM-M). During the experimental phase (6 d long), the four hays were grouped in combinations of two, resulting in six possible pairs (Table 3), with steers randomly assigned to receive different sequences of hay pairs. All hay pair combinations were offered daily to different steers so that, by the end of the 6-d feed preference trial, each animal received all pairs once.

**Table 3.** Mixed BFT–timothy grass hay combinations^*a*^ offered to beef steers during the feed preference trial

Pairs					
A	B	C	D	E	F
AM vs. AM-M	AM vs. PM	AM vs. PM-M	AM-M vs. PM	AM-M vs. PM-M	PM vs. PM-M

^*a*^AM = sunrise cut, nonmacerated; AM-M = sunrise cut, macerated; PM = sundown cut, nonmacerated; PM-M = sundown cut, macerated.

Hays within each pair were placed in individual partitions of the wooden mangers and offered to animals. It is important to note that the placement (left or right position) of individual hays of each pair in each manger’s partition was randomized. Chopped hay was weighed and delivered at 0800 h. The amount (DM basis) of hay offered daily was approximately 2.5% of BW (1.25% of BW of each hay within pair). The amount of hays offered and refused was recorded at 2, 4, 8, and 24 h postfeeding to calculate the rate of feed DM disappearance. Approximately 250 g of individual hays were sampled daily. These samples were weighed, oven-dried (55 °C, 48 h), ground through a 1-mm screen using a Wiley mill (Arthur H. Thomas Co.), and finally stored at room temperature until analyzed.

### Sample Analyses and Calculations

Samples of hay and feces from the digestibility and N balance and feed preference trials were analyzed for concentrations of DM (method 930.15; AOAC International, 2006), ash (method 942.05; AOAC, 1990), crude protein (CP; method 976.06; AOAC International, 2006), and ether extract [EE; Soxhlet extractor Soxtec 2050 (Foss Analytical, Hilleroed, Denmark) with hexane as solvent; method 920.39; AOAC, 1990]. Hay samples were further analyzed for soluble CP using borate phosphate buffer (Licitra et al., 1996). Samples of hay and feces were also analyzed for ash-free neutral detergent fiber (aNDFom; thermo-stable amylase-treated without sodium sulfite; Mertens et al., 2002), neutral detergent-insoluble CP (NDICP; Licitra et al., 1996), acid detergent-insoluble CP (ADICP; Van Soest et al., 1991), and total ethanol-soluble carbohydrates [TESC; 80:20 (vol:vol) ethanol:water extraction according to Hall et al. (1999) and Hall (2000)]. An Ankom^200^ fiber analyzer (Ankom Technology, Fairport, NY) was used for determinations of aNDF and acid detergent fiber (ADF). The ethanol-insoluble residue was analyzed for ash and CP (described above) and for starch concentration [Holm et al. (1986) as modified by Hall (2000)]. Neutral detergent-soluble fiber and organic acids were calculated as reported by Hall et al. (1999). Nonstructural carbohydrates were calculated as the sum of starch plus TESC (Hall et al., 1999). Fresh forage samples were analyzed for water-soluble carbohydrates (WSC) according to Dubois et al. (1956). The concentration of nonfiber carbohydrates (NFC) was calculated as described by Hall (2000) using the following equation:

$$\begin{array}{l} NFC\left(\%\right)=100-\left[ash\%+EE\%+CP\%+\left(aFDNom\%-NDICP\%\right)\right] \end{array}$$

Urine samples were thawed overnight at 4 °C and analyzed for total N (method 976.06; AOAC International, 2006) and urea N (diacetyl-monoxime with a Technicon autoanalyzer; industrial method no. 339-01; Technicon Instruments, Tarrytown, NY). Retained N was calculated as N intake subtracted from N excretion in feces and urine, all expressed in grams per day.

The concentration of total digestible nutrients (TDN) was calculated using the NRC (2001) equation as follows:

$$\begin{array}{ll} TDN\left(\%\right)=\;\; & dCP+daNDFom+dNFC\\ & +\left(2.25\times dEE\right) \end{array}$$

where *d*CP = apparent total tract digestibility of CP, *d*aNDFom = apparent total tract digestibility of aNDFom, *d*NFC = apparent total tract digestibility of NFC, and *d*EE = apparent total tract digestibility of EE. Digestible energy (DE) concentration of hays was calculated by multiplying each digestible nutrient by its respective calorimetric constant (NRC, 2001).

### Statistical Analysis

Intake and apparent total tract digestibility of nutrients, urinary excretion of nitrogenous compounds, and N balance data were analyzed as a 4 × 4 Latin square design with a 2 × 2 factorial arrangement of treatments using the MIXED procedure of SAS (SAS 9.4, SAS Inst. Inc., Cary, NC) according to the following statistical model:

$$\begin{array}{ll} Y_{ijkl}= & \;\;\mu +S_{i}+P_{j}+CT_{k}+MAC_{l}+CT\\ & \times MAC_{kl}+\varepsilon_{ijkl} \end{array}$$

where *Y*_ijkl_ is the observation for each dependent variable; μ is the overall mean; S_*i*_ is the random effect of the *i*th steer; *P*_*j*_ is the fixed effect of the *j*th period; CT_*k*_ is the fixed effect of the *k*th cutting time; MAC_*l*_ is the fixed effect of the *l*th maceration; CT × MAC_kl_ is the fixed effect of the interaction between the *k*th cutting time and *l*th maceration; and ε _ijkl_ is the residual error. The main effects of cutting time and maceration, and the cutting time × maceration interactions were tested using ANOVA. Significance was declared at *P* ≤ 0.05 and trends at 0.05 < *P* ≤ 0.10.

Dry matter intake of individual hays at each time postfeeding (i.e., 2, 4, 8, and 24 h) from both feed preference trials was averaged by animals across all tested pairs. Data were analyzed by the MIXED procedure of SAS (SAS 9.4), including cutting time and maceration as fixed effects and steer as the random effect. Significance was declared at *P* ≤ 0.05 and trends at 0.05 < *P* ≤ 0.10. Feed preference was analyzed by multidimensional scaling (MDS). Dry matter intake of each pair at 2, 4, 8, and 24 h postfeeding was used for delta (*δ*) calculation, which was done by subtracting the amount of DM consumed of the less preferred hay at a given time point from the amount of DM consumed of the most preferred hay divided by the sum of DMI of both hays (Buntinx et al., 1997; Fisher et al., 1999; Fisher et al., 2002). Delta data were analyzed by the MDS procedure of SAS (SAS 9.4) yielding a spatial arrangement of the tested hays in two dimensions.

## RESULTS AND DISCUSSION

### Forage Composition

During field drying, maceration increased moisture loss rate so that, immediately before tedding, the mean DM concentration of AM-M and PM-M forages was approximately 16% greater than the AM and PM counterparts (42.2% and 36.3% of fresh matter, respectively; Table 1). At baling, AM-M and PM-M hays had 7% greater DM concentration than AM and PM hays (Table 1). We expected a reduction in the catabolism of NSC by plant cells during field drying due to enhanced moisture losses in response to maceration. However, NSC concentration was similar between macerated and nonmacerated forages (Table 1). A mower-conditioner was used during harvesting in the present study, which may have offset or attenuated the differences in the rate of moisture losses between AM-M and PM-M versus AM and PM forages.

Forage CP concentration decreased (DM basis) from 14% to 11% between mowing and baling (Table 1), and it appears that maceration had a greater impact decreasing CP on sundown-cut than sunrise-cut hays. In fact, the average CP concentration of PM-M forage was 5% lower than that of PM forage at baling (11.5% vs. 12.1%, respectively). Crude protein reduction during field drying of legume sources is typically associated with leaf losses. Therefore, increased dehydration caused by maceration along with additional tedding likely increased leaf losses during wilting. The slightly greater fiber concentrations (i.e., aNDFom and ADF) at baling in AM-M and PM-M than AM and PM forages would support leaf losses because stems have more fiber and less CP than leaves (Hall et al., 1997). Even though maceration can decrease proteolysis by accelerating moisture losses, it may also stimulate proteolysis due to plant enzymatic action under wet weather conditions (Savoie, 2001). Thus, greater soluble CP concentration in macerated than nonmacerated hays may be associated with weather conditions prior to cutting (Table 2). Approximately 13.2 mm of precipitation occurred over a period of 3 d before cutting, during which daytime temperature and humidity averaged 17.6 °C and 79.7%, respectively (data not shown). Forages cut at sundown (PM and PM-M) had, on average, NSC concentration that was 17% greater than those cut at sunrise (AM and AM-M; 12.7% and 10.8%, respectively). At baling, PM and PM-M forages still had 17% greater NSC than AM and AM-M forages (10.4% and 8.8%, respectively).

Sugars analyzed as TESC or WSC were greater for PM and PM-M than AM and AM-M forages from mowing to baling (Table 1). Starch concentration decreased over time, with only 40% of the initial starch content remaining at baling (1.21% vs. 0.49%). Starch mobilization after cutting provides glucose to support plant cell respiration (Ruckle et al., 2017) and may explain its increased catabolism during field drying. In addition, it seems that starch degradation narrowed the difference in sugar concentrations between cutting times. Whereas starch concentration was 56% greater in PM and PM-M vesus AM and AM-M forages at mowing (Table 1), the difference in starch content between treatments narrowed at baling and in hay samples. Increased NSC accumulation by shifting cutting time from sunrise to sundown led to a 2-percentage unit reduction in aNDFom and ADF concentrations in hay samples (Table 2), likely caused by a dilution effect widely reported in the literature for fresh (Bertrand et al., 2008; Berthiaume et al., 2010) and conserved (Fisher et al., 2002; Brito et al., 2009) forages cut in the afternoon.

### Intake and Apparent Digestibility of Nutrients

Intake of DM did not differ and averaged 6.05 kg/d across treatments (Table 4). However, a cutting time by maceration interaction (*P* = 0.03) was observed for CP intake where maceration reduced CP intake by 18% in steers fed PM-M hay but not AM-M hay. This effect was possibly caused by a more pronounced reduction in CP concentration during field drying of PM-M versus AM-M hay due to the longer wilting time of hays cut at sundown compared with those cut at sunrise. Steers fed macerated hays consumed 13% less EE (*P* = 0.02), which can be explained by lower EE concentration in AM-M and PM-M versus AM and PM hays. Intakes of aNDFom, ADF, TESC, and NSC were not affected by cutting time or maceration (Table 4). Starch (*P* = 0.06) and NFC (*P* = 0.08) intakes tended to be affected by cutting time, with steers offered PM and PM-M hays consuming more starch and NFC than those receiving AM and AM-M hays. Increased starch and NFC intakes are consistent with the chemical composition of the forages fed, as hays cut at sundown had greater concentrations of starch and NFC than hays cut at sunrise.

**Table 4.** Intake and apparent total tract digestibility of nutrients in steers fed mixed BFT–timothy grass hays cut at sunrise or sundown macerated or not (*n* = 4 steers)

	Treatment^*a*^					*P*-value^*b*^		
Item	AM	AM-M	PM	PM-M	SEM	CT	MAC	CT × MAC
Intake^*c*^								
DM, kg/d	5.94	5.98	6.32	5.98	0.22	0.38	0.49	0.39
OM, kg/d	5.51	5.56	5.85	5.57	0.21	0.39	0.57	0.43
CP, kg/d	0.67^b^	0.65^b^	0.77^a^	0.64^b^	0.03	0.08	<0.01	0.03
EE, g/d	129	116	141	118	6.50	0.26	0.02	0.37
ADF, kg/d	2.54	2.50	2.56	2.51	0.10	0.84	0.56	0.96
aNDFom, kg/d	3.88	3.89	3.92	3.78	0.19	0.81	0.68	0.64
TESC, g/d	462	474	519	537	55.7	0.32	0.79	0.96
Starch, g/d	25.4	26.8	28.0	30.1	1.34	0.06	0.23	0.83
NSC,^*d*^ g/d	487	501	547	567	55.7	0.30	0.77	0.96
NFC,^*e*^ kg/d	1.05	1.12	1.28	1.24	0.09	0.08	0.86	0.57
Apparent digestibility, % of intake								
DM	64.6^b^	65.2^ab^	66.3^a^	64.7^b^	0.49	0.18	0.21	0.03
OM	67.5	67.8	69.1	67.4	0.52	0.19	0.15	0.06
CP	57.1^ab^	57.8^ab^	60.3^a^	54.6^b^	1.16	0.99	0.07	0.03
EE	33.1	24.6	40.0	34.8	4.13	0.03	0.07	0.62
ADF	59.8	59.4	59.4	58.3	0.70	0.30	0.31	0.64
aNDFom	66.7	66.5	67.5	65.0	0.71	0.67	0.11	0.14
TESC	97.1	97.0	97.0	97.5	0.27	0.37	0.35	0.17
Starch	69.0	68.5	69.0	71.3	2.16	0.53	0.69	0.55
NSC^*d*^	95.7	95.5	95.5	96.2	0.37	0.36	0.47	0.21
NFC^*e*^	78.0	80.4	78.3	80.7	1.85	0.88	0.23	0.98
TDN, % of DM	65.6^b^	65.7^b^	67.3^a^	65.2^b^	0.47	0.19	0.07	0.04
DE, Mcal/kg of DM	2.84^b^	2.85^b^	2.93^a^	2.82^b^	0.02	0.21	0.05	0.04

^*a*^AM = sunrise cut, nonmacerated; AM-M = sunrise cut, macerated; PM = sundown cut, nonmacerated; PM-M = sundown cut, macerated.

^*b*^CT = cutting time effect; MAC = maceration effect; CT × MAC = cutting time by maceration interaction effect.

^*c*^Feed intake restricted to 90% of ad libitum.

^*d*^TESC + starch.

^*e*^NFC = 100 − [CP + EE + (aNDFom − NDICP) + ash].

^ab^Least square means with different superscripts differ at *P* ≤ 0.05.

Maceration by cutting time interactions (*P* = 0.03) were found for the apparent total tract digestibilities of DM and CP (Table 4). Specifically, maceration decreased DM and CP digestibilities in steers fed the PM-M hay but had no effect on those receiving the AM-M counterpart. Sunrise- and sundown-cut hays were mechanically macerated 4 and 12 h after cutting, respectively. It is conceivable that the longer wilting time of PM-M versus AM-M hay led to greater shattering loss of leaves and decreased DM and CP digestibilities, as leaves have greater CP and lower neutral detergent fiber (NDF) concentrations than stems (Hall et al., 1997). In addition, a trend for maceration by cutting time interaction (*P* = 0.06) was observed for the apparent total tract digestibility of OM, which was mostly caused by the interaction in CP digestibility (Table 4). In our previous dual-flow continuous culture study, a significant cutting time by maceration interaction was detected such that maceration decreased the true digestibilities of DM and OM of mixed BFT–timothy grass hay cut at sundown but increased these variables in sunrise-cut hay (Kokko et al., 2013). Contrarily, Kokko et al. (2013) observed no treatment effects on the apparent digestibility of CP. Berthiaume et al. (2010) reported greater apparent DM digestibility for high- versus low-NSC alfalfa herbage without concomitant changes in NDF, ADF, and CP digestibilities in dual-flow continuous culture fermentors. Ether extract total tract digestibility was less in steers fed AM and AM-M than PM and PM-M hays (*P* = 0.03), and maceration tended to decrease EE digestibility (*P* = 0.07). However, EE digestibility was low (mean = 33.1%) in the present study, suggesting that endogenous fat may have accounted for most of the fecal output of EE. Therefore, these observed changes in the apparent total tract digestibility of EE have limited biological implications because EE intake is typically low when forages are fed as the sole feed source.

Maceration and cutting time had no effect on the apparent total tract digestibilities of aNDFom, ADF, and NFC. Brito et al. (2009) also observed no effect of cutting time on DM, OM, and NDF digestibilities (% of intake) in the rumen and total gastrointestinal tract. Kokko et al. (2013) detected a significant cutting time by maceration interaction, with maceration reducing ADF digestibility from 66.1% to 52.9% in mixed BFT–timothy grass hay cut at sundown but increasing it from 47.4% to 49.8% when cutting at sunrise. They also observed that hays cut at sundown had greater NDF digestibility than those cut at sunrise (Kokko et al., 2013). Discrepancies between in vitro and in vivo results are not uncommon and reflect the inability of in vitro systems to account for physiological processes like absorption of end products of fermentation, chewing, rumination, and hormonal effects on nutrient metabolism.

Maceration by cutting time interactions (*P* = 0.04) were found for both TDN and DE concentrations (Table 4), indicating that maceration decreased fermentable energy in PM-M hay but no effect in AM-M hay. According to Savoie (2001), maceration may improve the nutritive value of hay by speeding up the drying process. However, maceration can also increase leaf losses due to mechanical shattering, ultimately reducing CP and increasing NDF concentrations (Savoie, 2001). The detrimental effect of maceration on TDN and DE contents of PM-M hay may have been further exacerbated by differences in wilting time as discussed previously. Despite AM-M and PM-M hays being macerated at the same time, hays cut at sundown had longer wilting time than those cut at sunrise, which may have led to increased leaf losses and compromise of nutritive value.

### Nitrogen Balance

A cutting time by maceration interaction was observed for N intake (*P* = 0.03; Table 5). Fecal N excretion, expressed in grams per day, did not differ across treatments, but a cutting time by maceration interaction was detected for fecal N excretion as a proportion of N intake (*P* = 0.03; Table 5). Specifically, while maceration increased fecal N excretion with feeding PM-M hay, no effect was detected when steers were offered AM-M hay. However, a potential dilution effect caused by metabolic fecal N losses may have contributed to the greater apparent total tract digestibility of CP from PM versus PM-M hay. A cutting time by maceration interaction was observed for absorbed N, expressed as grams per day (*P* = 0.01) or as a proportion of N intake (*P* = 0.03), thus indicating that maceration decreased absorbed N in steers fed the PM-M hay but had no effect on those receiving the AM-M counterpart. Previous studies revealed that absorbed N was greater in steers fed sundown- versus sunrise-cut grasses (Huntington and Burns, 2007, 2008), but this response was not uniform across forage species. While beef steers fed switchgrass (*Panicum virgatum* L.) baleage cut at sundown had increased absorbed N compared with those fed the sunrise-cut counterpart (55.6% vs. 52.3% of N intake), no diurnal cutting management effect on absorbed N was detected in steers fed gamagrass (*Tripsacum dactyloides* L.) baleage (Huntington and Burns, 2007).

**Table 5.** Nitrogen balance in steers fed mixed BFT–timothy grass hays cut at sunrise or sundown macerated or not (*n* = 4 steers)

	Treatment^*a*^					*P*-value^*b*^		
Item	AM	AM-M	PM	PM-M	SEM	CT	MAC	CT × MAC
N intake, g/d	107.7^b^	103.6^b^	123.5^a^	101.8^b^	4.48	0.08	<0.01	0.03
N output, absorbed, and retained, g/d								
Fecal	46.4	43.9	48.9	46.4	1.92	0.24	0.23	0.99
Absorbed	61.3^b^	59.7^b^	74.6^a^	55.4^b^	3.37	0.11	<0.01	0.01
Urinary	41.8	41.1	42.2	34.3	3.35	0.27	0.14	0.21
Manure (feces + urine)	88.2	85.0	91.1	80.7	3.70	0.79	0.03	0.19
Apparent retained	19.5	18.6	32.4	21.0	2.79	0.02	0.04	0.07
N output, absorbed, and retained, % of N intake								
Fecal	42.9^ab^	42.2^ab^	39.7^b^	45.4^a^	1.16	0.99	0.07	0.03
Absorbed	57.1^ab^	57.8^ab^	60.3^a^	54.6^b^	1.16	0.99	0.07	0.03
Urinary	39.0	39.5	33.4	34.1	2.59	0.05	0.79	0.96
Manure (feces + urine)	81.9	81.7	73.1	79.5	2.33	0.03	0.17	0.15
Apparent retained	18.1	18.3	26.9	20.5	2.33	0.03	0.17	0.15
Apparent retained N, % of absorbed N	31.2	31.7	44.7	37.5	4.16	0.03	0.33	0.39
Urinary urea N, g/d	33.5	29.5	31.5	28.0	3.90	0.67	0.37	0.95
Urinary urea N, % of N intake	31.1	28.6	24.6	28.2	3.47	0.36	0.87	0.41
Urinary urea N, % of total urinary N	79.7	72.3	72.5	82.1	6.66	0.85	0.86	0.24

^*a*^AM = sunrise cut, nonmacerated; AM-M = sunrise cut, macerated; PM = sundown cut, nonmacerated; PM-M = sundown cut, macerated.

^*b*^CT = cutting time effect; MAC = maceration effect; CT × MAC = cutting time by maceration interaction effect.

^ab^Least square means with different superscripts differ at *P* ≤ 0.05

The total amount of N excreted in urine (grams per day) was not affected by the cutting time or maceration in the current experiment (Table 5). However, urinary N excretion, as a proportion of N intake, was reduced by shifting the cutting time from sunrise to sundown (*P* = 0.05). This reduction in urinary N excretion observed in steers fed PM and PM-M hays may be explained by the increased uptake of NH_3_-N by ruminal microbes. Previous research revealed that increasing NSC concentration of alfalfa baleage or alfalfa herbage improved microbial protein synthesis in vivo (Brito et al., 2009) and in vitro (Berthiaume et al., 2010). Although steers fed sundown-cut hays had only numerically greater NSC intake than those fed sunrise-cut hays (+13%), we believe that this difference is relevant, as pointed out by others who found a similar difference between PM and AM forages and better N use efficiency in cows fed PM-cut red clover baleage (Antaya et al., 2015) or PM-allocated perenial ryegrass herbage (Chen et al., 2017). In addition, we observed in our companion paper that fermentors dosed with mixed BFT–timothy hays cut at sundown had decreased ruminal NH_3_-N concentration and tended to increase microbial N flow (Kokko et al., 2013).

Urinary excretion of urea N, expressed in grams per day (mean = 30.6 g/d), proportion of total N intake (mean = 28.1%), or total urinary N excretion (mean = 76.6%) was not affected by treatments (Table 5). This lack of difference in urinary urea N excretion between treatments agrees with previous studies (Huntington and Burns, 2008; Sauvé et al., 2009). Although the amount of manure N excretion (fecal N plus urinary N) was reduced by maceration (*P* = 0.03), no difference between macerated and nonmacerated treatments was observed when manure N excretion was expressed as a proportion of N intake. Decreased manure N excretion (grams per day) in steers fed AM-M and PM-M hays has marginal practical implications because neither fecal N nor urinary N outputs were affected by maceration in the present experiment.

The amount of N apparently retained in bodily tissues increased by shifting cutting time from sunrise to sundown (*P* = 0.02). Specifically, steers retained 7.7 g more N daily when fed PM and PM-M than AM and AM-M hays. In a literature review, Kohn et al. (2005) reported that 26 g of N was retained per kilogram of BW gain in growing cattle. Therefore, this additional 7.7 g of N retained in steers fed sundown-cut hays could be translated in 294 g greater daily BW gain compared with steers receiving sunrise-cut hays. According to the Beef Cattle Nutrition Requirement Model (NASEM, 2016), protein accounts for 18.4% of the gain in an Angus steer weighing 325 kg of BW and gaining 0.6 kg/d, indicating that 29.4 g of N is deposited in the body for each kilogram of gain. Therefore, the extra 7.7 g of retained N could be equivalent to 262 g of daily gain. Using the empirical level of solution in the NASEM (2016), we also estimated dietary metabolizable energy and metabolizable protein supply (an average nutrient composition was used). Estimated metabolizable energy and metabolizable protein supply allowed ADG of 0.609 and 0.438 kg/d, respectively, indicating that when feeding our experimental hays, protein but not energy appears to be limiting animal growth. It can be hypothesized that an increase in absorbed N would likely improve the growth rate. Apparent retained N, as a proportion of N intake, was greater with feeding sundown- versus sunrise-cut hays (*P* = 0.03). This indicates that changing cutting time from sunrise to sundown improved N utilization, thus in agreement with elevated milk N efficiency (milk N/N intake) and milk true protein yield in dairy cows fed baleages made from alfalfa (Brito et al., 2008) or timothy grass (Brito et al., 2016) cut at sundown, respectively. Furthermore, Huntington and Burns (2007) observed that retained N was increased (+16 g/d) when beef steers were fed switchgrass baleage cut at sundown versus sunrise. In the current study, increased N use efficiency in steers fed sundown- versus sunrise-cut hays was evidenced by improved retained N as a proportion of absorbed N.

Maceration decreased (*P* = 0.04) the amount of apparent retained N in the body of steers in the present study (−6.15 g/d; Table 5), thus suggesting that AM-M and PM-M hays could promote a decrease of 236 g/d in ADG based on Kohn et al. (2005) or 209 g/d based on NASEM (2016). It is important to emphasize that this maceration effect on retained N was consistent with decreased N intake in steers fed PM-M hay as discussed earlier. However, the lack of maceration effect on apparent retained N, as a proportion of absorbed N, is consistent with similar N use efficiency in steers fed macerated and nonmacerated hays.

### Feed Preference

Previous research showed that increasing dietary sucrose from 3% up to 10% (DM basis) increased DMI linearly in lactating dairy cows (Broderick et al., 2008). In addition, a positive correlation (*r* = 0.94) between NSC concentration and forage DMI was reported for different ruminant species (Fisher et al., 1999). Gregorini et al. (2006) observed that beef heifers exhibited more intense grazing when allocated to herbage mid-afternoon (1500 h) than early morning (0700 h). Likewise, Huntington and Burns (2007) demonstrated that beef steers fed gamagrass or switchgrass baleage cut at sundown (mean = 30.4% NSC) spent more time eating and had greater DMI than those fed baleage made from these same grass species cut at sunrise (mean = 19% NSC). Overall, these results suggest that increased dietary concentration of sugars or NSC stimulates feed intake.

**Figure 1.**
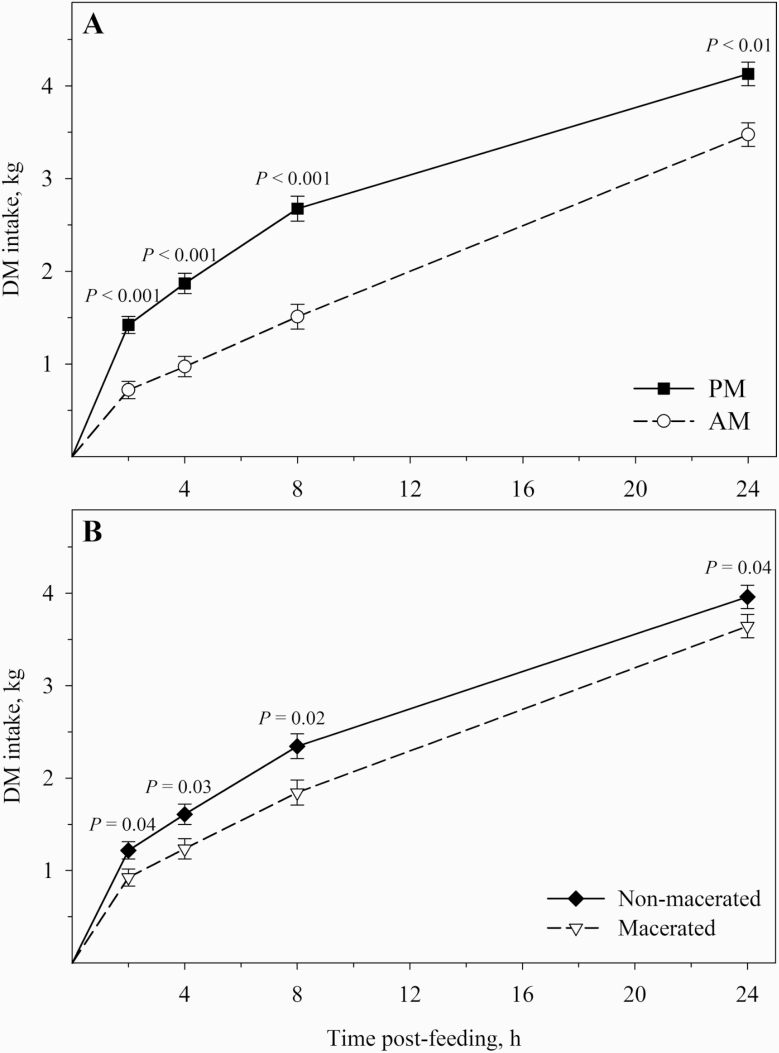
Dry matter intake postfeeding in beef steers fed mixed BFT–timothy grass hay cut at sunrise or sundown (A) macerated or not (B). No interaction effect between cutting time and maceration was observed; *n* = 6 steers.

We observed no treatment by time postfeeding interaction for feed intake rate in the present study. Therefore, the main effects of cutting time and maceration were presented separately (Fig. 1). Although DMI was not affected by the cutting time in the digestibility trial (Table 4), when hays were offered in pairs during the feed preference trial, steers chose PM and PM-M hays over AM and AM-M hays. However, hays were restricted to 90% of ad libitum intake during the digestibility trial. As shown in Fig. 1A, the rate of DMI of sundown-cut hays in the first 8 h postfeeding was about twice that observed for sunrise-cut hays (*P* < 0.01). Our results are consistent with previous reports showing that beef steers prefer forage cut at sundown due to the greater NSC concentrations compared with forages cut at sunrise (Fisher et al., 1999; Huntington and Burns, 2007). Despite the small difference in TESC concentration between sunrise-cut (mean = 8.2%) and sundown-cut hays (mean = 9.4%), cattle are able to detect changes in sugar concentration as low as 1.25% (DM basis) according to Mayland et al. (2000).

Maceration (AM-M and PM-M hays) decreased intake rate (*P* < 0.05), thus suggesting that steers preferred nonmacerated hays (AM and PM) as shown in Fig. 1B. However, the effects of maceration on beef cattle intake are controversial. In a review paper, Savoie (2001) reported that DMI in beef cattle may be positively, negatively, or not affected by mechanical maceration. These contradictory results may be a consequence of the effect of maceration on hay quality, which can be positive or negative (Savoie, 2001). In the present study, maceration negatively affected both hay nutritive value and feed intake rate.

**Figure 2.**
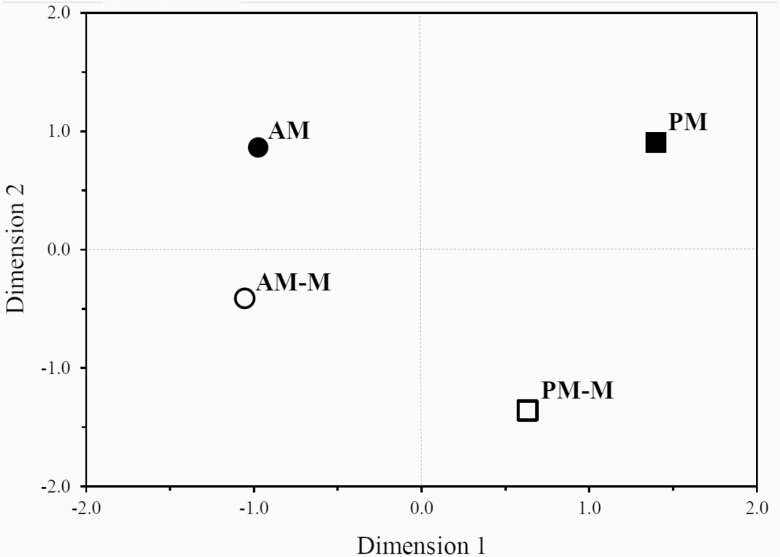
Multidimensional scaling showing feed preferences in beef steers offered mixed BFT–timothy grass hay cut at sunrise or sundown macerated or not. The most preferred hay is in the upper right corner and the least preferred hay in the lower left corner. AM = sunrise cut, nonmacerated; AM-M = sunrise cut, macerated; PM = sundown cut, nonmacerated; PM-M = sundown cut, macerated; *n* = 6 steers.

Multidimensional scaling has been used as a tool to graphically represent feed preference in studies conducted with ruminants (Buntinx et al., 1997; Fisher et al., 2002). The coordinates obtained with MDS are adjusted to show preferred treatments in the upper right corner and least preferred treatments in the lower-left corner. Therefore, PM hay was the most preferred forage source, whereas the AM-M hay was the least preferred by steers (Fig. 2). In our MDS analysis (Fig. 2), the effect of cutting time was represented along dimension 1 axis with PM and PM-M hays showing a positive value, while AM and AM-M hays a negative value. Increased preference for PM and PM-M hays may be explained by elevated NSC concentration as it has been demonstrated that NSC was the determinant factor for forage preference in ruminants (Mayland et al., 2000). Nonmacerated hays (AM and PM) had positive values for dimension 2, while macerated hays (AM-M and PM-M) showed negative values. The greater distance between PM versus PM-M hays compared with AM versus AM-M hays suggests that maceration had a more distinct influence on feed preference of sundown- than sunrise-cut hays (Fig. 2). Overall, our MDS results imply that steers were able to discriminate between hay sources.

## CONCLUSION

Our data indicated no synergistic effects of cutting time and maceration on hay nutritive value under the conditions of the present study. Feeding mixed BFT–timothy grass cut at sundown and harvested as hay with increased NSC concentration improved apparent retained N and feed preference in growing crossbred Angus steers. The slight increase in moisture loss due to maceration was not enough to prevent NSC use during field drying. Maceration also decreased the concentration of CP and increased that of fiber in mixed BFT–timothy grass hays, leading to reduced feed preference and N retention. Nutrient digestibility, N balance, and feed preference data should be interpreted cautiously due to the small number of animals used in the experiments. Further research with larger sample size is needed to better understand the impact of maceration on feed preference and N utilization in beef cattle fed forage-only diets.
